# Ancient coins: cluster analysis applied to find a correlation between corrosion process and burial soil characteristics

**DOI:** 10.1186/1752-153X-6-S2-S9

**Published:** 2012-05-02

**Authors:** Rita Reale, Susanne H Plattner, Giuseppe Guida, Maria Pia Sammartino, Giovanni Visco

**Affiliations:** 1 Chemistry Dept, "La Sapienza" University, p.le A. Moro, 5, 00185 Rome, Italy; 2 Istituto Superiore per la Conservazione ed il Restauro (ISCR), Via di San Michele, 23 00153 Roma, Italy

## Abstract

Although it is well known that any material degrades faster when exposed to an aggressive environment as well as that "aggressive" cannot be univocally defined as depending also on the chemical-physical characteristics of material, few researches on the identification of the most significant parameters influencing the corrosion of metallic object are available.

A series of ancient coins, coming from the archaeological excavation of Palazzo Valentini (Rome) were collected together with soils, both near and far from them, and then analysed using different analytical techniques looking for a correlation between the corrosion products covering the coins and the chemical-physical soil characteristics. The content of soluble salts in the water-bearing stratum and surfacing in the archaeological site, was also measured.

The obtained results stress the influence of alkaline soils on formation of patina. Cerussite, probably due to the circulation of water in layers rich in marble and plaster fragments, was the main corrosion product identified by X-ray Diffraction (XRD). Copper, lead and vanadium were found in soil surrounding coins. By measuring conductivity, pH and soluble salts content of the washing solutions from both coins and soils, we could easily separate coins coming from different stratigraphic units of the site.

Data were treated by cluster and multivariate analysis, revealing a correlation between part of the coins and the nearby soil samples.

## Introduction

Coins are important evidence in terms of history, art and economy. Besides the composition of the alloy, what is usually revealed by written documents appears in form of effigies, short inscriptions and symbols useful. Taking into account what above said, it can be surely stated that coins are particular and important findings during archaeological investigations as source of documentation, understanding and knowledge of mankind evolution. However, coins are also test samples of metal very sensitive to the effect of deterioration processes, even more if intensified by polluted environments. Their state of conservation, obviously enough, depends also on normal wearing processes before burial as well as on all chemical-physical changes of the burial environment [1,2]. Obviously, specific information is not available, but recent studies confirm that deterioration of buried metallic objects sensibly accelerated during the last 50-100 years [3,4], as a result of the technological progress, to the point that some authors deem as secondary, while evaluating corrosion, whether a bronze object was buried for 300 or 3000 years [5].

Preservation of buried coins is mainly affected by a limited set of variables, that are those favouring corrosion [6]. A moderately well aerated and moist soil, for example sand reached by the water table, the pores exposed to cyclical exposure to water and oxygen, would promote a strong corrosion of metals/alloys [7,8]. In contrast, a fine-grained sediment with abundant clay or a coarse-grained one rich in gravel should be less harmful, because aeration and humidity would be seldom balanced.

Acidic and salt-rich sediments would also favour the corrosion of metals. While low pH values normally cause thermodynamic instability of the outer corrosion layers (oxides and hydroxides), high salt contents would provide highly conductive electrolytes.

In order to obtain information on the corrosion process on buried coins, we used non invasive and mini-invasive analytical techniques on a series of Roman bronze coins (from Palazzo Valentini, Rome, Italy) and associated soil samples, taken both near and far from the find spots of the coins, but anyhow in the same archaeological area [9].

In a preliminary research, 23 coins had been studied with scarce results due to a defective sampling procedure and to a very bad state of conservation and consequent impossibility of numismatic identification.

As a second step, we studies 12 coins and associated sediments coming from the same site but excavated in March 2009. The innovative part of the present research lies in the sum of complementary analytical techniques applied, in the particular pre-treatment of soil samples and in the statistical data treatment.

## Results

Analyses of coins and soil samples by Scanning Electron Microscopy coupled to Energy Dispersive Spectroscopy (SEM/EDS) detected both typical soil elements (Mg, Al, Si, P, Na, K, S, Ti) and alloy constituents [10]. In particular copper and lead were found in all coins while tin and arsenic were detected frequently. Moreover, vanadium was present in the patina of all coins, while the same element occurred only in some cases on the soil samples, gathered both far and near the analyzed coins.

Energy Dispersive X-ray Fluorescence analysis (ED-XRF) confirmed the relatively high lead content of the coin's alloy. Comparing the results with those of SEM/EDS analysis, arsenic was not detected, but small percentages of silver are on record. Both techniques found tin in low percentage values, except for sample Pt8-US1012, which showed ca.10% of this metal.

Fig. C in Additional file 1 shows the SEM image of coin A14 US10H enhancing a growth of globular crystals rich in lead and vanadium. In the lower part of figure the EDS spectra of the interest zone is also shown.

XRD tests frequently identified cerussite (PbCO_3_) and litharge (PbO) [11]. Copper was detected in form of cuprite (Cu_2_O) and tenorite (CuO) [9] (data available in Table 6 of ADF). The abundance of cerussite may be explained by the alkaline characteristics of the archaeological layers, clearly detected by pH measurements. Soil alkalinity is certainly due to the many fragments of carbonatic rocks and plaster rubble dispersed in the burial soil and affected by circulating water solutions.

Conductivity and salt content measurements of both water from the tanks where coins were washed and the soil samples did not allow absolute quantitative information, for the lack of standard reference data; however the information was used for internal comparison and correlation analysis with a positive outcome.

In this section we show only a summary of results obtained by statistic data analysis; original analytical data are instead summarized in charts and tables in section "Description of additional data file".

All analytical data were put in a matrix, obtaining five layers. Two sub matrixes were extracted and separately analysed.

In order to highlight trends and/or similarities among our objects, we drew scatter plots representing values relative to each column obtained in each of the matrices A and B as sum of three of the original five layers.

**Figure 1:**
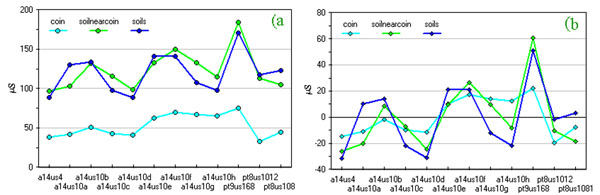
**Conductivity values of coin washing solutions a) original data; b) data after column centring by subtraction of the mean column value**.

In Figure 1a conductivity values show a difference between data coming from the washing solutions of coin's patina and soils; the latter ones being significantly higher.

Matrix pre-treatment, generally applied as first step of Exploratory Data Analysis (EDA), can alter data structure and thus seriously biasing interpretation [12]. After column centring by subtraction of the mean column value of each matrix layer, one obtains the new graph in Figure 1b. This simple way of treating the data reveals, better than the raw analytical values, a correlation between solutions coming from the washing of coins and near-to-coin soil samples. On the contrary, the original data better represented absolute analytical values, very similar for soil samples and significantly lower in coins.

**Figure 2:**
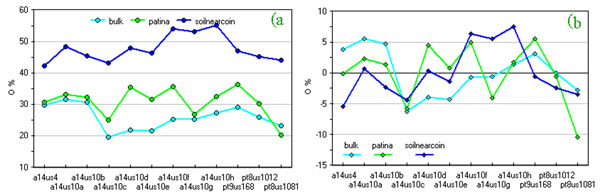
**Oxygen content (%) of coins, their patinas and surrounding soils**. a) original data; b) data after column centring by subtraction of the mean column value.

**Figure 3:**
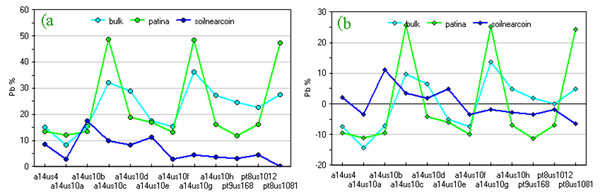
**Lead content (%) of coins, their patinas and surrounding soils**. a) original data; b) data after column centring by subtraction of the mean column value.

Applying the same procedure on compositional data we looked for a set of expected correlation. For example, in Figure 2(a,b) and Figure 3(a,b) oxygen and lead percentages of coin bulk, patina and soil near coins are compared. Absolute values are in the expected order while correlation is less evident than in the previous case.

**Figure 4:**
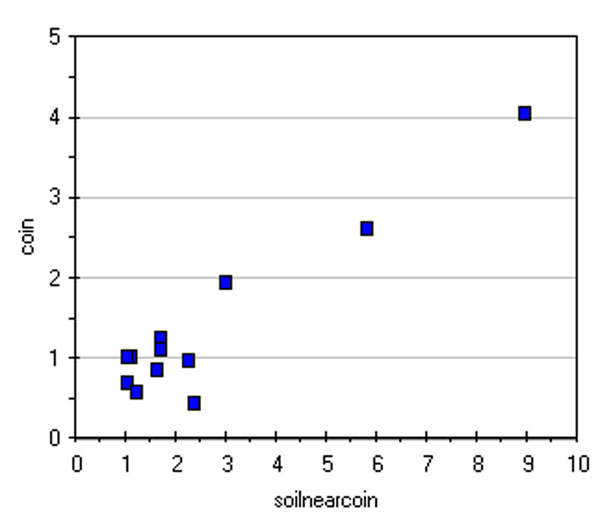
**Scatter plot of the ${SO}_{4}^{2-}$ content (ppm) of coin washing solutions and extracting solutions of near-to-coin soil samples**.

**Figure 5:**
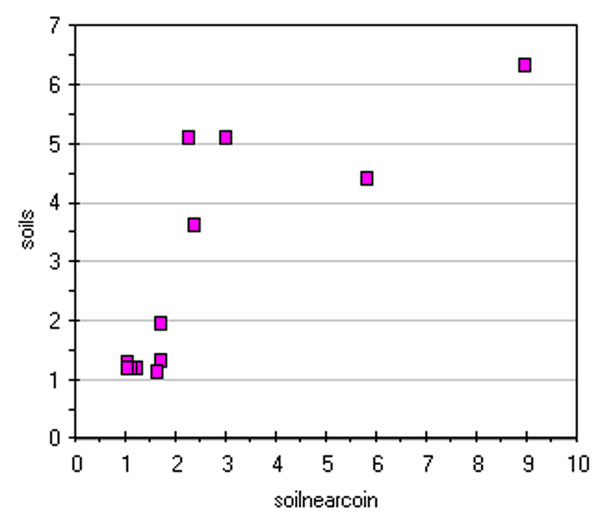
**Scatter plot of the ${SO}_{4}^{2-}$ content (ppm) of extracting solutions of near-to-coin and far-from-coin soil samples**.

As an example of the treatment of chromatographic data, Figure 4 shows a scatter plot of the SO_4_^-2 ^content found in the solutions coming from the washing of coins and near-to-coin soil samples; the same plot is shown in Figure 5 for soils far from and near the coins. In both cases a positive correlation is observed.

**Figure 6:**
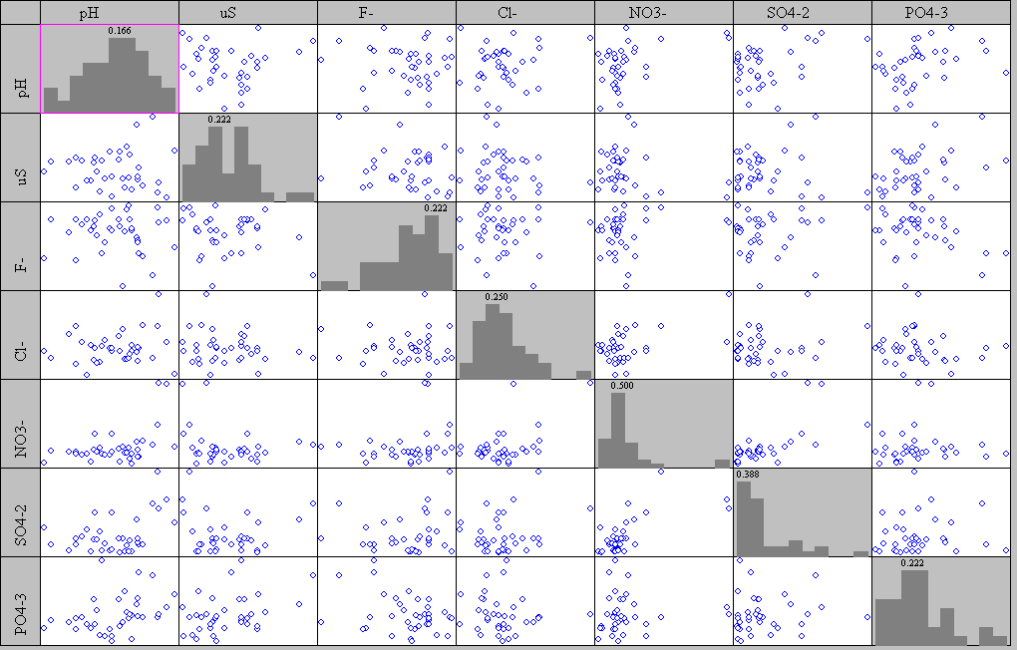
**Scatter plot of matrix A, anions, pH, conductivity, after column centring**.

The scatter plot of matrix A, after column centring is shown in Figure 6. As no evident correlation was found, all the relative variables can be used for the successive data treatment.

**Figure 7:**
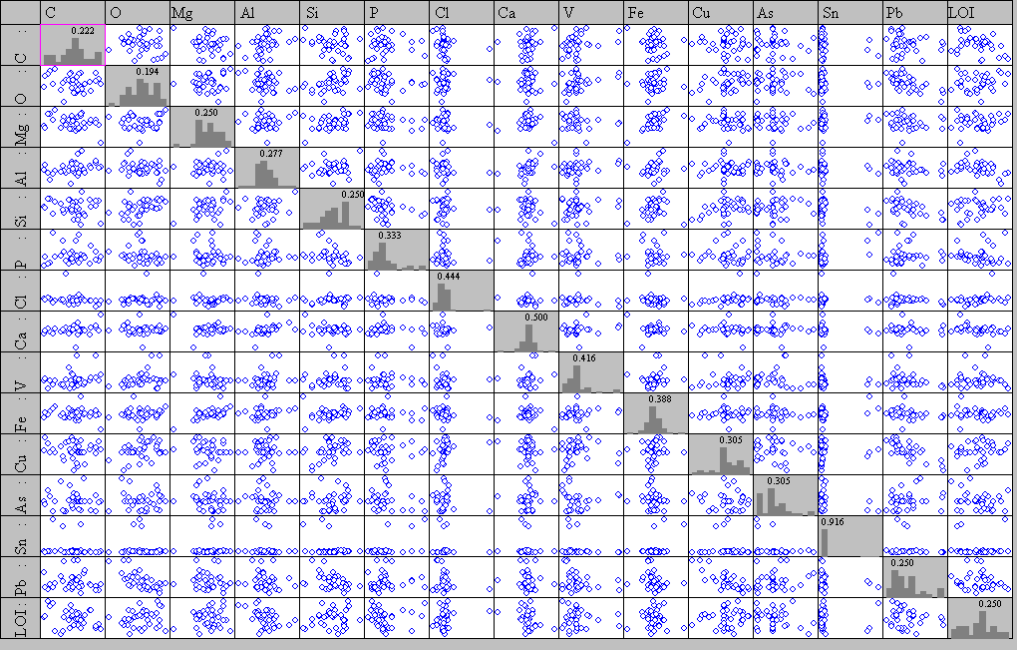
**Scatter plot of matrix obtained by EDS data, main elements**.

Figure 7 shows that also for data coming from the SEM/EDS analyses no evident correlation exists. Scatter plot matrixes show outliers for Sn and Cl; original data reveals that Sn outliers come from coin pt8-1012, the only one with a high Sn content, while the Cl^- ^outlier is due to a high content of this element in the patina of coin 14-us10b.

In order to obtain full information from all the measured variables, we decided to implement a multivariate statistic analysis.

**Figure 8:**
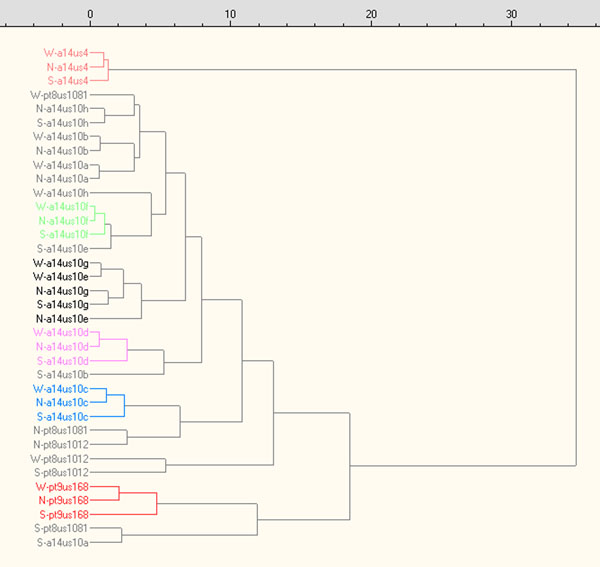
**Dendrogram obtained by HCA using pH, μS, F^-^, Cl^-^, NO^3-^, ${SO}_{4}^{2-}$, ${PO}_{4}^{3-}$ data (coin washing solutions and extracting solutions of near-to-coin and far-from-coin soil samples)**.

The complete five layer matrix is available in .XLS format (version 97) in the ADF. Figure 8 shows the dendrogram obtained by HCA for the seven variables (pH, μS, F^-^, Cl^-^, NO^3-^, SO_4_^-2^, PO_4_^-3^) measured in the washing solutions of coins (W) and soils samples near to (N) and far (S) from coins. In five cases we obtained grouping clusters evidenced by colours.

Furthermore, the dendrogram enhances strong similarities between burial soil samples and coins (i.e. W-a14us10b/N-a14us10b) thus fully confirming the effect of the soil chemistry on the composition of patinas.

**Figure 9:**
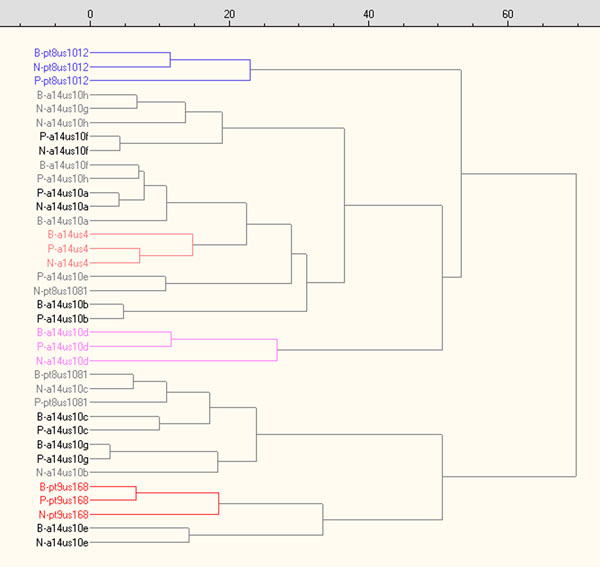
**Dendrogram obtained by EDS elemental composition data of coin bulk, patina and soil near coin**.

An analogous dendrogram appears in Figure 9 for the elemental composition of coin bulk (B), patina (P) and soil near to coin(N); four triplets are evidenced by colours, three of which refer to the same coins already identified by the previous dendrogram.

Among the measured variables iron was excluded, due to its normally high concentrations in soils and concentration levels around the LOD in the bulk of coins. Unfortunately, the coins' bulk composition analysis is affected by the patinas, because, even for analytical purposes, these latter could not be removed as due to obvious ethical problems, the artefacts could not be scraped and radically cleaned. Furthermore, the use of the rodent 4 mm HSS iron wheel may have changed the iron content of the cleaned surface patches of the coins.

In Figure 9 one also identify 6 couples, with similarities between soil samples and patinas (NP) or soil and coin bulks (NB) that must be considered of the same importance or higher than the triplets. As a further example of the validity of this approach, Figure 8 evidenced in black groups two coins (with the exclusion of a far-soil), 14us10g and 14us10e, which were found one near the other.

## Conclusion

When archaeologists find in a dig a coin or another interesting findings, their first interest is to use them to date the associated layers or the context.

This immediate interest sometimes results in poor sampling strategies affecting the successive analytical procedure.

This is why in the present study we could use only 12 coins; really, for the first series of coins the corresponding near and far soil samples lack so being no useful for a meaningful statistical treatment of archaeological [13] and analytical data. However, as the chosen analytical techniques coupled to the multivariate data treatment revealed meaningful correlations between the coins, their patina and the surrounding micro-environment, the first steps of this research are quite encouraging. It must be taken into account that, due to the different kind of data and overall to their big mole, a traditional univariate data treatment would be very difficult even in the case of a low number of samples.

## Experimental

A complete analytical procedure is reported in Figs. A and B of Additional file 1 for coin and soil samples respectively.

### Statistical analyses

Chemometry allows to individuate similarity among similar or different objects. Aim of our data treatment was to search for similarity among coins, their patina and the surrounding soil, while search of similarity among coins goes beyond the aim.

The Hierarchical Cluster Analysis (HCA), after a suitable pre-treatment of the data matrix, was used as Exploratory Data Analysis.

Variables in the matrix (see ADF) are put in columns; each one refers to a chemical element identified by compositional analyses of coins or to a measured parameter such as pH, conductivity, etc.

Objects, i.e. coins, are put in the matrix rows [14], plus the one specifying the column labels.

Better results were obtained with a 3D matrix containing 5 layers: the first one contains data from analyses of washing solutions of soils sampled far from coins; the second one reports analogous data for soils surrounding coins (burial soil) plus elemental composition data; data of patina analyses filled the third and fourth layer and refer to the washing solutions and the elemental composition respectively; the last layer lists the chemical elements constituting the coin bulk.

Obviously, only the second layer is complete, as it was possible to analyse both the solid phase (elemental composition of the insoluble matter) and the washing solutions (data on the soluble matter).

The first column of each layer contains the alphanumerical label defined by the archaeologists.

The data matrix could contain even more data, but we choose to construct a completely filled matrix avoiding statistic methods like Least Square Estimation [14] or other robust methods [15] for filling of empty matrix cells.

The less invasive pre-treatment method useful to enhance the difference among objects is probably the column centring that can be obtained by subtracting the arithmetic average of the column from each value in the same column. A discussion about the use of other "central values" like median, geometric mean, harmonic mean, mode, and so on goes beyond the aim of this paper.

By autoscaling or column centring we removed the numerical difference among variables (as an example, in our case from pH to Cu concentration) allowing a self organization of the data in clusters using a simple method for distance and agglomeration.

Generally pre-treatment like autoscaling or centring is done after the matrix unfolding but we preferred to do scaling on the original matrix (5 layers) and then divide it in two new matrices. The first one includes the unfolded layers 1,2 and 3, where variables are data coming from the analyses of the washing solutions; the column number remained 7 (plus the label one) while rows increased to 3 × 12 (plus the label one). In the same way we obtained the second matrix joining layers 2, 4 and 5 (data coming from the elemental analyses of the solid phases). Obviously, in the new matrices, data from the original layer 2, exceeding the variables present in the other two, were eliminated.

The HCA was used to find similarities among objects; it works as a bottom-up procedure based on the distances among objects: the first cluster is assigned to the two nearest objects, the second one is assigned to other two of the remaining objects showing the least distance and so on.

As our hypothesis bases on the effect of the soil characteristics on the corrosion process of the coins, we expected to obtain "a series" of clusters, equal to the number of coins, each one connected to bigger clusters as a function of the different soil characteristics.

All data were stored using the software Lotus 123 ver. 9.8, the same software was used to obtain all the average values calculated from measurements on different points of each coin, to draw graphs of Figure 2 and similar ones, to construct the two matrix MatA and MatB and to export data in .xls format.

Datalab software was used for all the statistical calculations on the matrix; data scaling, HCA by different agglomerative methods and distances as well as to draw the dendrograms; it is an evolution of Inspect [16] that, in the free version, is sufficient to treat small matrices such as ours (A and B).

The distance values, in HCA, can be strongly affected by differences in scale among the dimensions from which the distances are calculated. We chose a simple classic method for scaling and we did the unfolding process after the studentisation on layers, i.e. on the columns of the original matrix.

The only method we used to calculate distances for the HCA is the squared Euclidean distance; it doesn't use the typical Euclidean root square, which in such cases could alter values, in particular it can increase them with the strongest effect on the greatest distance. (*You may want to square the standard Euclidean distance in order to place progressively greater weight on objects that are further apart*, by R.L. Carter [17].

For the Cluster Analysis, among all possible grouping methods, we used Average Linkage (*In average linkage the distance between two clusters is the average distance between pairs of observations, one in each cluster*, by SAS online documentation) [18] for the matrix A and Complete Linkage (*In complete linkage, the distance between two clusters is the maximum distance between an observation in one cluster and an observation in the other cluster*, by SAS online documentation [18] for the matrix B.

### Sampling

Coins were found during the excavation carried out under Palazzo Valentini, which is located in the centre of Rome, very close to the Forum Traianum. Both coins and soils were sampled in nine different stratigraphic units (SU) located in three different rooms (room 14, PT9 and PT8) at an height ranging from 0 to -1.7 m from the building cellar, corresponding to a mean deep of about 7 m from the street level.

Soils far from the coins but coming from the same SU were sampled in order to obtain data not influenced by the contact coin-soil.

In order to improve the analytical knowledge of the site, the water-bearing stratum surfacing in the archaeological site was sampled in two different points inside the same rooms.

### Pre-treatment

Coins were washed by ultrapure water (20 mL) in a Elmasonic S30H ultrasonic bath for 10 min and then pH and conductivity were measured. The procedure was repeated until a constant conductivity value was measured for three successive washings; this criterion was adopted to establish a complete cleaning and desalting of the coins. The total content of salts was successively determined by IC. Taking into account the ethic rules of restoration, the corrosion layers were removed from coins by means of a dental piezo-electric scaler *Titanus^®^E (controlled by a microprocessor, 50/60 Hz, 21 VA, **f**requency: 28000 ÷ 31000 H*z). Removed corrosion layers were then prepared for XRD analysis i.e. ground in a agate mortar and layered on a suitable emery glass holders.

Before EDXRF and SEM/EDS analysis three small areas of the coin was more deeply cleaned by rotary rodents tools in steel irons.

Soil samples were dried in oven at 105 °C for half an hour and then sieved at 2 mm; 0.6 g were pressed at 10 atm for 15 min, using a Perkin Elmer 12T hydraulic laboratory press (0-12 ton), to obtain pastilles for SEM-EDS and XRF analysis. Other sieved portions were ground in an agate mortar; aliquots of 250 mg were put in 25 mL deionised water and treated in a Elmasonic S30H ultrasonic bath to extract soluble salts for the IC analysis.

### SEM/EDS analyses

Electronic microscopy coupled to X-ray microanalysis (SEM-EDS) was carried out by means of a Zeiss Evo 60.

Instrument setting was optimised for each single analysis [19] looking for "compromise" conditions ensuring the best analysis quality by all the three detectors.

For a preliminary view of sample's morphology and distribution of the corrosion patina a low magnitude image (70x) by secondary electrons (SE) was used; basing on this, the most interesting and representative zones were then explored under higher zoom conditions [20].

Distribution maps of the average atomic density were obtained as image by the backscattered electrons (BSE), enhancing the heavier elemental composition of the patina compared to that of the alloy.

On interest points of both zones, with and without patina, EDS microanalysis was performed on single grains and crystals or on small areas (to identify the average elemental composition).

The same procedure was carried out for soil samples in order to identify common elements in soil and coins so obtaining data useful to search for any correlation by the successive multivariate analysis.

In total 78 measurements were carried out: 29 on cleaned coin areas, 28 on coin patina, 18 on soil samples.

### XRF analyses

A portable EDXRF was used for X-ray fluorescence measurements (35 KVolt, Amptek X-Ray detectors XR-100CR, Amptek, Multichannel Analyzer MCA 8000A).

The X-ray tube was set at tension and current equal to 36 KV and 0.12 mA respectively; 300 s was adopted as exposure time.

The obtained spectra were processed by McUiICR software and for quantitative analysis the function single peak area - 2 Gauss was used. Further 7 different reference standards were used.

### XRD analyses

For X-ray diffraction analysis (XRD) a Seifert MZ IV with copper anticathode (λCuKα = 1.542 Å) was used, applying a tension of 40 KV and a current of 30 mA. Scan angle 2θ was set between 3° and 70° with a 0.02θ sampling step and 2 s exposure time.

### IC analyses

The salt content of coin washing solutions, soil extracts and groundwater samples was determined by using a Metrohm 761 Compact IC equipped with a Metrosep A Supp4 column (61006430 - Size: 5.0 × 250 mm Particle size: 9.0 μm) and a 20.0 μL loop.

Eluent was prepared starting from sodium hydrogencarbonate (NaHCO_3_) by Sigma (purity certified by producer: 99.7-100.3%) and sodium carbonate (Na_2_CO_3_) by Merck, (purity certified by producer: 99.88% +/- 0.05%); the final concentration was 1.7 mmol/L and 1.8 mmol/L respectively. Deionised water (0.8 μS conductivity) was obtained by means of a Millipore Direct-Q 3 UV deionizer and used for sample and reagent preparation. First of the analysis, samples were treated in a Universal 32R centrifuge (by Hettich Zentrifugen) at 4000 rpm, for half an hour at 4°C; successively they were filtered through disposable membranes Minisart, 0.2 μm in PTFE (by PBInternational).

### pH and conductivity measures

A pH Meter pH211 Hanna Instruments equipped with a combined glass electrode was used for the pH measurements.

For conductivity measurements a Primo 5 Hanna Instrument equipped with a cell with internal microprocessor for automatic calibration and temperature compensation was employed.

## Competing interests

None of the authors received any financing, scholarship, contract or consulting from societies mentioned in this paper. Instruments were bought regularly through national research funding.

## Authors' contributions

Among the authors we would like to differentiate RR, who graduated with a thesis focused on the presented research. The remaining authors contributed to the presented research in a different manner but at the same level.

## Authors' information

RR, SHP, MPS, GV: Rome University, La Sapienza, Mat. Phy. Natural Science Faculty, Rome, Italy

GG: ISCR, High Institute for Conservation and Restoration, Rome, Italy

## Supplementary Material

Additional file 1 **All analytical data was put in an external data file**. The file includes the complete used five layer matrix before the unfolding; an additional layer (6) reports some other values measured but not used in this work (pH of the bearing stratum water and so on). Figures A and B describe, as flow charts, the analytical procedures adopted to obtain data relative to both coins and soils. Figure C shows, as an example, the SEM image and the EDS spectra of one of the coin. Tables 1-3 list some information on coins such as the finding location in the archeological site, image, dimensions and so on. Tables 4-5 list the main physical characteristics of soils while table 6 summarises the corrosion products found by XRD. Click here for file
