# Supplementary material for: Ancient coins: cluster analysis applied to find a correlation between corrosion process and burial soil characteristics
Source: Chem Cent J. 2012 May 2;6(Suppl 2):S9. doi: 10.1186/1752-153X-6-S2-S9 (PMC3342128; doi:10.1186/1752-153X-6-S2-S9)
Supplement: Additional file 1 — All analytical data was put in an external data file. The file includes the complete used five layer matrix before the unfolding; an additional layer (6) reports some other values measured but not used in this work (pH of the bearing stratum water and so on). Figures A and B describe, as flow charts, the analytical procedures adopted to obtain data relative to both coins and soils. Figure C shows, as an example, the SEM image and the EDS spectra of one of the coin. Tables 1-3 list some information on coins such as the finding location in the archeological site, image, dimensions and so on. Tables 4-5 list the main physical characteristics of soils while table 6 summarises the corrosion products found by XRD. [file 1752-153X-6-S2-S9-S1.ZIP › R-Reale_(Ancient-Coins-Cluster-Analysis-Applied-to)_Suppl-tab-fig.doc]

| **Table 1.** Coins location | | |
| --- | --- | --- |
| **Site** | **Stratigraphic Unit** | **Depth* (m)** |
|  |  |  |
| **PT 9** | SU 168 | 0 |
| **PT 8** | SU 1012 | 1.5 |
|  | SU 1078 | 1.2 |
|  | SU 1081 | 0.35 |
| **PT 14** | SU 4 | 1.5 |
|  | SU 8 | 1.7 |
|  | SU 10 a | 0.6 |
|  | SU 10 b | 0.6 |
|  | SU 10 c | 0.2 |
|  | SU 10d | 0.6 |
|  | SU 10e | 1.1 |
|  | SU 10f | 1.1 |
|  | SU 10g | 0.35 |
|  | SU 10h | 0.35 |
|   **PT 14**  **PT 9**  **PT 8** | | |
| * measured from bulding cellar floor level | | |

| **Table 2** Coins catalogue | | | | |
| --- | --- | --- | --- | --- |
| **N** | **ID number (find)** | **Image of Coin** | **Diameter (mm)** | **Weight (g)** |
|  |  |  |  |  |
| **1** | **A14 SU10a**  **AE3** |  | Mean **19.48** | 3.22 |
|  |  |  | Stand. Dev. **0.02** |  |
| **2** | **A14 SU10b**  **AE3** |  | Mean **18.41** | 3.23 |
|  |  |  | Stand. Dev. **0.03** |  |
| **3** | **A14 SU10c**  **AE3** |  | Mean **18.59** | 1.77 |
|  |  |  | Stand. Dev. **0.12** |  |
| **4** | **A14 SU10d**  **AE4** |  | Mean **14.29** | 2.41 |
|  |  |  | Stand. Dev. **0.05** |  |
| **5** | **A14 SU10e**  **AE3** |  | Mean **17.23** | 1.97 |
|  |  |  | Stand. Dev. **0.03** |  |
| **6** | **A14 SU10f**  **AE3** |  | Mean **18.70** | 2.43 |
|  |  |  | Stand. Dev. **0.15** |  |
| **7** | **A14 SU10g**  **AE3** |  | Mean **17.14** | 2.94 |
|  |  |  | Stand. Dev. **0.02** |  |
| **8** | **A14 SU10h**  **AE4** |  | Mean **16.43** | 2.48 |
|  |  |  | Stand. Dev. .**0.06** |  |
| **9** | **A14 SU4**  **AE3** |  | Mean **17.35** | 1.76 |
|  |  |  | Stand. Dev. **0.01** |  |
| **10** | **PT9 SU168**  **AE4** |  | Mean **13.67** | 1.30 |
|  |  |  | Stand. Dev. **0.03** |  |
| **11** | **PT8 SU1012**  **AE4** |  | Mean **11.98** | 1.07 |
|  |  |  | Stand. Dev. **0.03** |  |
| **12** | **PT8 SU1081**  **AE4** |  | Mean **16.28** | 2.50 |
|  |  |  | Stand. Dev. **0.04** |  |

| **Table 3** *Main coins characterisation* | | | | | |
| --- | --- | --- | --- | --- | --- |
| ID number (find) | weight before washing (g) | weight after washing (g) | Δ weight (g) | diameter (mm) | volume (mm^3^) |
| **A14 10a** | 3.220 | 3.210 | 0.004 | 19.48 | 595.77 |
| **A1410b** | 3.230 | 3.220 | 0.005 | 18.41 | 532.12 |
| **A14 10c** | 1.770 | 1.770 | 0.003 | 18.59 | 461.19 |
| **A14 10d** | 2.410 | 2.410 | 0.005 | 14.29 | 352.66 |
| **A14 10e** | 1.970 | 1.970 | 0.004 | 17.23 | 466.09 |
| **A14 10f** | 2.430 | 2.430 | 0.007 | 18.70 | 439.21 |
| **A14 10g** | 2.940 | 2.930 | 0.007 | 17.14 | 461.23 |
| **A14 10h** | 2.480 | 2.470 | 0.007 | 16.43 | 339.05 |
| **A14 4** | 1.760 | 1.670 | 0.099 | 17.35 | 460.79 |
| **Pt9 168** | 1.300 | 1.300 | 0.003 | 13.67 | 161.36 |
| **Pt8 1012** | 1.070 | 1.070 | 0.001 | 11.98 | 191.53 |
| **Pt8 1081** | 2.500 | 2.490 | 0.004 | 16.28 | 416.11 |

| **Table 4** Main soils characteristics | | | |
| --- | --- | --- | --- |
| Stratigraphic Unit (SU) | soil type | Humidity  content (%) | Temp. (°C) |
| **A14 SU8** | sand/clay | 28.3 | 21.6 |
| **A14 SU10** | sand/clay | 22.5 | 21.5 |
| **A14 SU10** | sand/clay | 26.4 | 19.4 |
| **A14 SU10** | sand/clay | 27.67 | 21.4 |
| **A14 SU4** | sand/clay | 23.75 | 21.7 |
| **PT9 SU168** | sand | 27.14 | 20.1 |
| **PT8 1078** | sand/clay | 21.7 | 19.3 |
| **PT8 1081** | sand/clay | 28.16 | 19.4 |
| **PT8 1012** | sand/clay | 23.3 | 20.1 |

| **Table 5** Characterisation of soils by Munsell chart | | | | |
| --- | --- | --- | --- | --- |
|  | | | | |
| **ID number (find)** | | **Value** | **Chroma** | **Hue Y/R** |
| ***PT9 169*** |  | 5 | 4 | 7.5 |
| ***Amb 14***  ***SU 4*** |  | 4 | 4 | 7.5 |
| ***PT8 1081*** |  | 3 | 2 | 10 |
| ***PT8 1012*** |  | 2 | 1 | 7.5 |
| ***Amb 14***  ***SU 10*** |  | 3 | 5 | 10 |

| **Table 6** Corrosion products identified by XRD (X-ray diffraction) | | | | | | | | |
| --- | --- | --- | --- | --- | --- | --- | --- | --- |
| ID number (find) | Anorthite  (CaAl_2_Si_2_O_8_) | Calcite  (CaCO_3_) | Cerussite  (PbCO_3_) | Cuprite  (Cu_2_O) | Litharge  (PbO) | Mottramite  (PbCu(VO_4_)  (OH) ) | Quartz  (SiO_2_ ) | Tenorite  ( CuO) |
| **A14 SU10a** |  | X | X |  |  |  | X |  |
| **A14 SU10b** |  | X | X |  |  |  | X |  |
| **A14 SU10c** |  | X | X |  |  |  | X |  |
| **A14 SU10d** | X* | X | X | X |  |  | X |  |
| **A14 SU10e** | X* | X | X |  |  |  | X |  |
| **A14 SU10f** |  | X | X |  |  |  | X |  |
| **A14 SU10g** |  | X | X |  |  |  |  |  |
| **A14 SU10h** | X* | X | X |  |  |  | X |  |
| **A14 SU4** |  | X | X |  | X |  |  | X |
| **Pt9 SU168** |  | X |  |  |  | X* | X |  |
| **Pt8 SU1012** |  | X | X |  |  |  | X |  |
| **Pt8 SU1081** |  |  | X |  |  |  |  |  |
| * Plausible | | | | | | | | |

**Figure c,** SEM analysis of coin A14 SU10H – above: zoom 1000x enhancing growth of globular crystals rich in lead and vanadium; below: EDS spectra of zone of interest.

**Figure a,** Flow chart of the analytical procedure used in the coins analysis

**Figure b,** Flow chart of the analytical procedure used in the soil analysis
